# Dynamic Analysis and Pattern Visualization of Forest Fires

**DOI:** 10.1371/journal.pone.0105465

**Published:** 2014-08-19

**Authors:** António M. Lopes, J. A. Tenreiro Machado

**Affiliations:** 1 Institute of Mechanical Engineering, Faculty of Engineering, University of Porto, Porto, Portugal; 2 Institute of Engineering, Polytechnic of Porto, Porto, Portugal; Universidad de Zarazoga, Spain

## Abstract

This paper analyses forest fires in the perspective of dynamical systems. Forest fires exhibit complex correlations in size, space and time, revealing features often present in complex systems, such as the absence of a characteristic length-scale, or the emergence of long range correlations and persistent memory. This study addresses a public domain forest fires catalogue, containing information of events for Portugal, during the period from 1980 up to 2012. The data is analysed in an annual basis, modelling the occurrences as sequences of Dirac impulses with amplitude proportional to the burnt area. First, we consider mutual information to correlate annual patterns. We use visualization trees, generated by hierarchical clustering algorithms, in order to compare and to extract relationships among the data. Second, we adopt the Multidimensional Scaling (MDS) visualization tool. MDS generates maps where each object corresponds to a point. Objects that are perceived to be similar to each other are placed on the map forming clusters. The results are analysed in order to extract relationships among the data and to identify forest fire patterns.

## Introduction

Forest fires are a major concern in many countries, like United States, Australia, Russia, Brazil, China and Mediterranean Basin European regions [1]–[3]. Every year forest fires consume vast areas of vegetation, compromising ecosystems and contributing to the carbon dioxide emissions that are changing Earth's climate [4]–[5]. Besides the long-term economic implications associated to the climate change, forest fires have direct impact upon economy due to the destruction of public and private property and infrastructures [6]. Fires are mainly caused by natural factors, human negligence, or even human intent. Fire propagation and burnt area depend on many natural factors and conditions, not only on the terrain orography and the type of vegetation, but also on the efficacy of detection and suppression strategies. Moreover, fires caused by incendiaries contribute to increase the complexity of the phenomena. Understanding the underlying patterns of forest fires in terms of their size and spatiotemporal distributions may help the decision makers to take preventive measures beforehand, identifying possible hazards and deciding strategies for fire prevention, detection and suppression [7]–[8].

Forest fires have been studied using classical statistical tools. However, those methods reveal limitations, both in capturing all characteristics underneath forest fires dynamics, and the evolution along years [9]. Forest fires dynamics exhibits correlations in size, space and time. Size-frequency distributions unveil long range memory, which is typical in complex systems. Correlation between data is characterized by self-similarity and absence of characteristic length-scale, meaning that forest fires exhibit power-law (PL) behaviour [10]–[13].

Several studies have been published during the last years about this topic [14]–[17]. In references [18]–[19] it is shown that forest fires exhibit PL frequency-size relationship over many orders of magnitude and that such behaviour seems consistent with the self-organized criticality arising in complex systems. The most important practical implication of such results is that the frequency-size distribution of small and medium fires can be used to quantify the risk of large fires [19]. Nevertheless, some authors [15] suggest that a simple PL distribution of sizes may be too simple to describe the distributions of forest fires over their full range.

In reference [20] the time dynamics of forest fires is investigated and it is shown that forest fires exhibit time-clustering phenomena. More recently, the fractality of the forest fires was addressed in [21] using spatial and temporal fractal tools. The authors prove that these phenomena exhibit space–time clustering behaviour.

In this paper we look at forest fires from the perspective of dynamical systems. A public domain forest fires catalogue containing data of events occurred in Portugal, in the period 1980 up to 2012, is addressed. The data is analysed in an annual basis, modelling the occurrences as a sequence of Dirac impulses. Therefore, instead of modelling individual forest fires, we are describing the global dynamics along several decades. In this perspective, mutual information and visualization trees, generated by hierarchical clustering algorithms, are used. The Multidimensional Scaling (MDS) tool is adopted in order to compare and to extract relationships among the data. Finally, we propose an amplitude-space embedding technique that produces a clear fire pattern classification.

## Characterization of the Dataset

Data from forest fires is available online at the Portuguese Institute of Nature and Forest Conservation (INCF), http://www.icnf.pt/portal/florestas/dfci/inc/estatisticas, and the catalogue contains events since 1980 up to 2012. Ignitions might have different sources, as natural causes, human negligence or human intentionality, among others. The data analysed in this paper was retrieved in December, 2013. Each record contains information about the events date, time (with one minute resolution), geographic location and size (in terms of burnt area). We decided to discard small size events, as those are prone to measurement errors, by adopting a cutoff threshold value of *A_min_* = 10 hectares.

**Figure 1:**
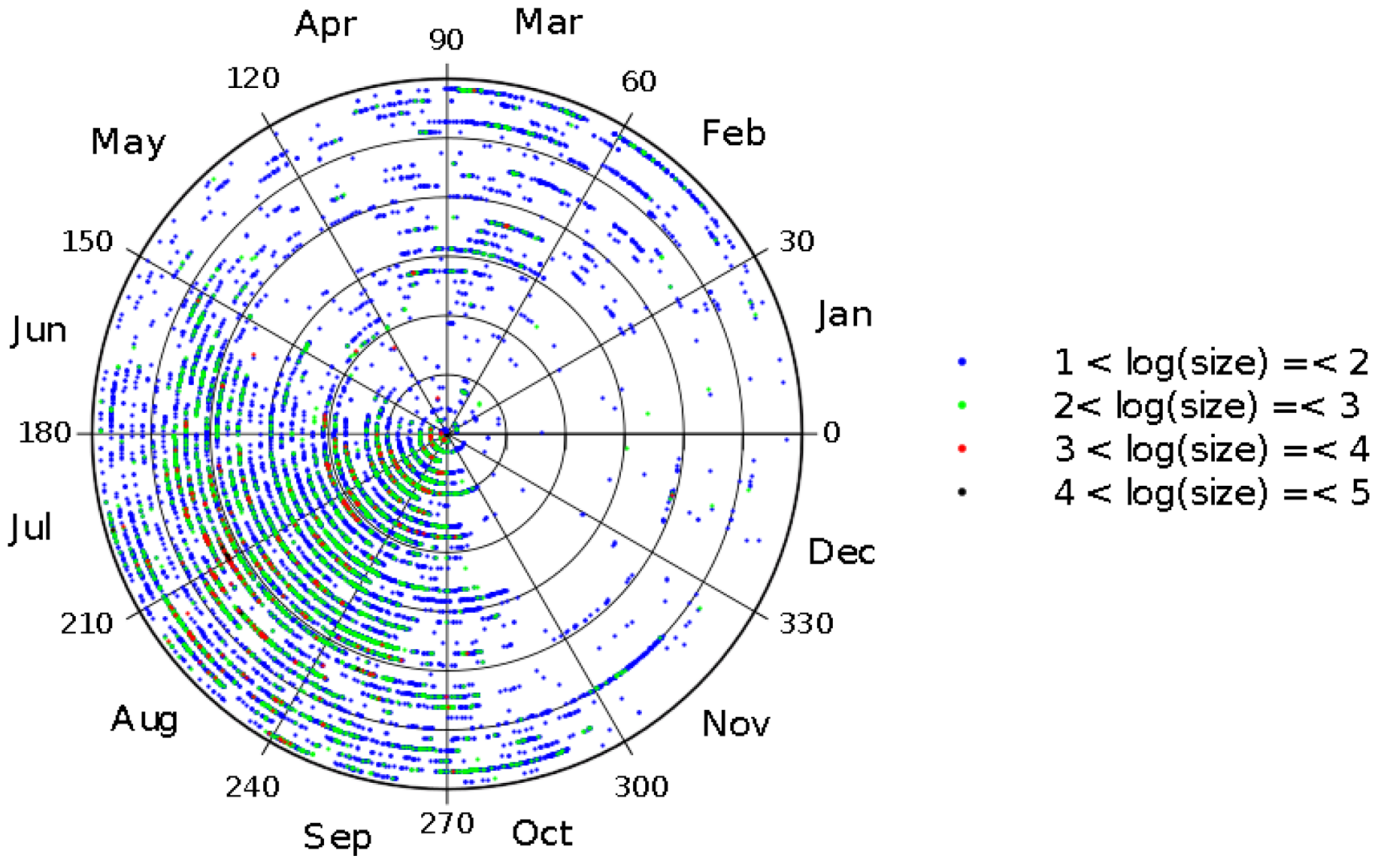
Temporal evolution and size (in log units) of forest fires registered in Portugal in the time period 1980–2012, with burnt area larger than *A_min_* = 10 ha. Each (*r*, *θ*) point represents the time of the event and the color represents the size.

Fig. 1 illustrates the temporal evolution and size of the events occurred in Portugal, during 1980–2012 and meeting the cutoff threshold criterion. We tackle the concept of ‘circular time’ (since there is a kind of one-year periodicity, with December close to January and not the opposite, as a Cartesian scale implicitly assumes). The (circular) time scale evolves along an Archimedean spiral, with origin at the center of the circumferences, given by:
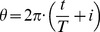
(1)

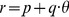
(2)where (*r*, *θ*) denotes the radius and angle coordinates, respectively, *i* = 0, …, 32, represents the year and *p* = *q* = 1. The burnt area is expressed in logarithmic units and is related to the color of the marks. We can note two annual cycles: the first is weaker and includes the months of February and March; the second is stronger and is due to the major incidence of fires during summer [22].

**Figure 2:**
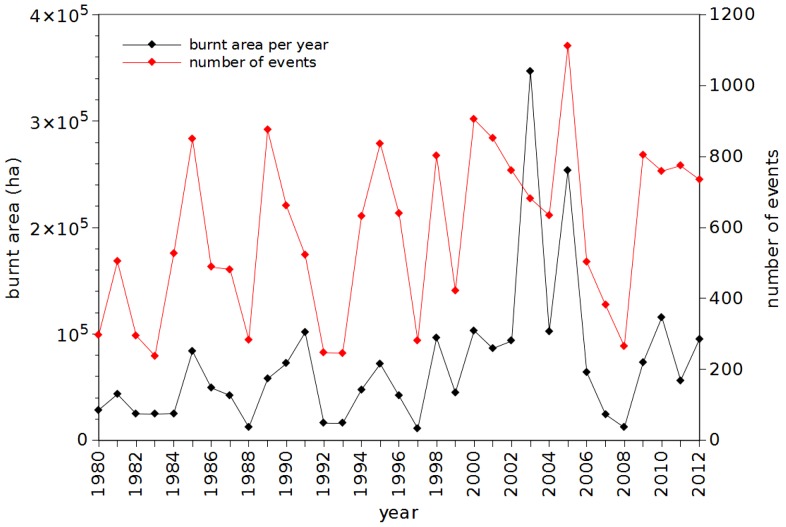
Evolution of burnt area per year and number of occurrences registered in Portugal in the time period 1980–2012 (are considered events with burnt area larger than *A_min_* = 10 ha).

**Figure 3:**
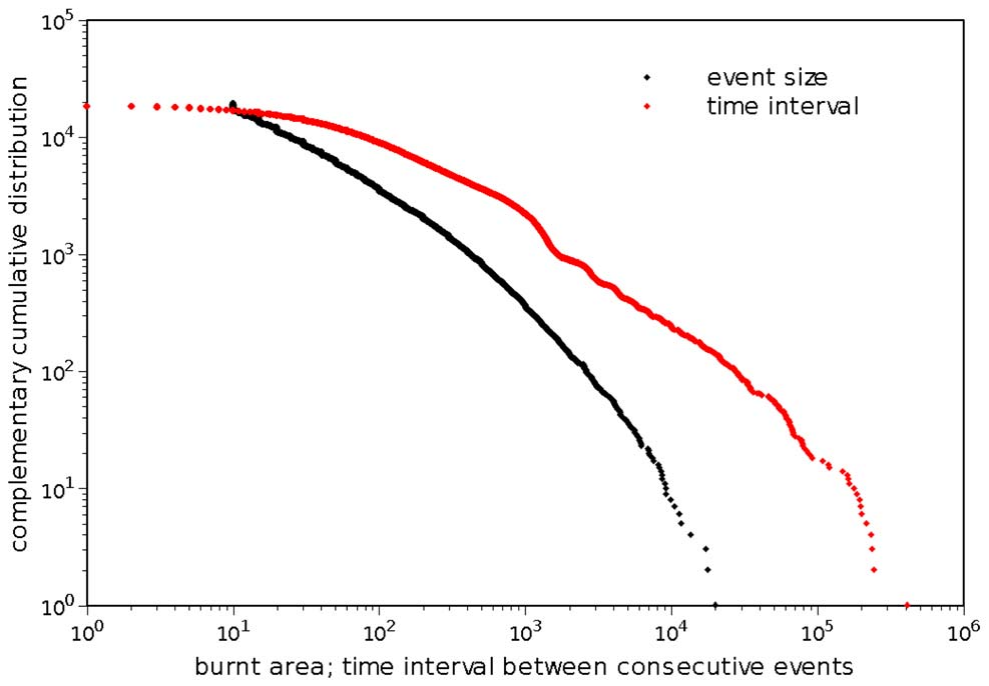
Complimentary cumulative distribution of events size and time interval (in minutes) between consecutive events, corresponding to occurrences registered in Portugal in the time period 1980–2012, with burnt area larger than *A_min_* = 10 ha.

In Fig. 2 we depict the evolution of the burnt area per year and number of occurrences versus year. It is visible the increasing number of events as well as the strong activity verified around the middle of the decade 2000–2009. Nevertheless, the charts reveal a large volatility and pose difficulties to capture some trend. We observe minimal values for years 1983, 1988, …, 2008, and maximum values for 2003 and 2005, but no straightforward method to correlate data points. Fig. 3 represents the complementary cumulative distributions of the events size and the time interval between consecutive events.

The results shown above illustrate through simple statistics the increasing importance of understanding the behavior of forest fires and characterizing the spatiotemporal distributions unveiled by such a complex phenomenon. For that purpose, in the next sections we adopt several complementary mathematical tools.

## Mutual Information Analysis

In this section we adopt the mutual information to correlate forest fires annual patterns. First we compute the mutual information, based on events size (i.e., burnt area), for each pair of years in the time period 1980–2012. Second, we use a hierarchical clustering algorithm to find relationships among the data. Visualization trees are used to highlight the interpretation of the results.

### Mutual Information

The mutual information is a measure of the statistical dependence between two random variables, giving the amount of information that one variable “contains” about the other. If *X_i_* and *X_j_* are two discrete random variables, then the mutual information, *I*(*X_i_*, *X_j_*), is given by:
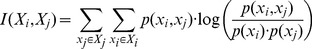
(3)where *p*(*x_i_*, *x_j_*) is the joint probability distribution function of (*X_i_*, *X_j_*), and *p*(*x_i_*) and *p*(*x_j_*) are the marginal probability distribution functions of *X_i_* and *X_j_*, respectively.

The concept of mutual information comes from the information theory [23] and has been adopted in the study of complex systems from diverse fields, namely in experimental time series analysis, in DNA and symbol sequencing and in providing a theoretical basis for the notion of complexity [24]–[30].

In this section, instead of expression (3), we use the normalized mutual information, *I_N_*(*X_i_*, *X_j_*), given by [31]:
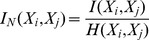
(4)where *H*(*X_i_*, *X_j_*) represents the joint entropy between *X_i_* and *X_j_*:
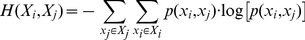
(5)


The normalized mutual information *I_N_*(*X_i_*, *X_j_*) ∈ [0, 1] simplifies comparison across different conditions and improves sensitivity.

Forest fires are analysed in an annual basis. For each year, *i* = 0, …, 32, in the period 1980–2012 the events are represented by:
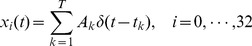
(6)leading to 33 one-year length time series. This means that the events are modelled as Dirac impulses, where *A_k_* represents fire size (i.e., burnt area), *t_k_* is the instant of occurrence (with one minute resolution), *t* represents time and *T* denotes the time period of one year.

The signals *x_i_*(*t*) are then normalized according to (7):
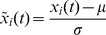
(7)where *μ* and *σ* represent the mean and standard deviation values of all events listed during 1980–2012, with magnitude larger than *A_min_* = 10 ha. The mutual information is calculated to correlate events occurred in different years of the analysed time period.

**Figure 4:**
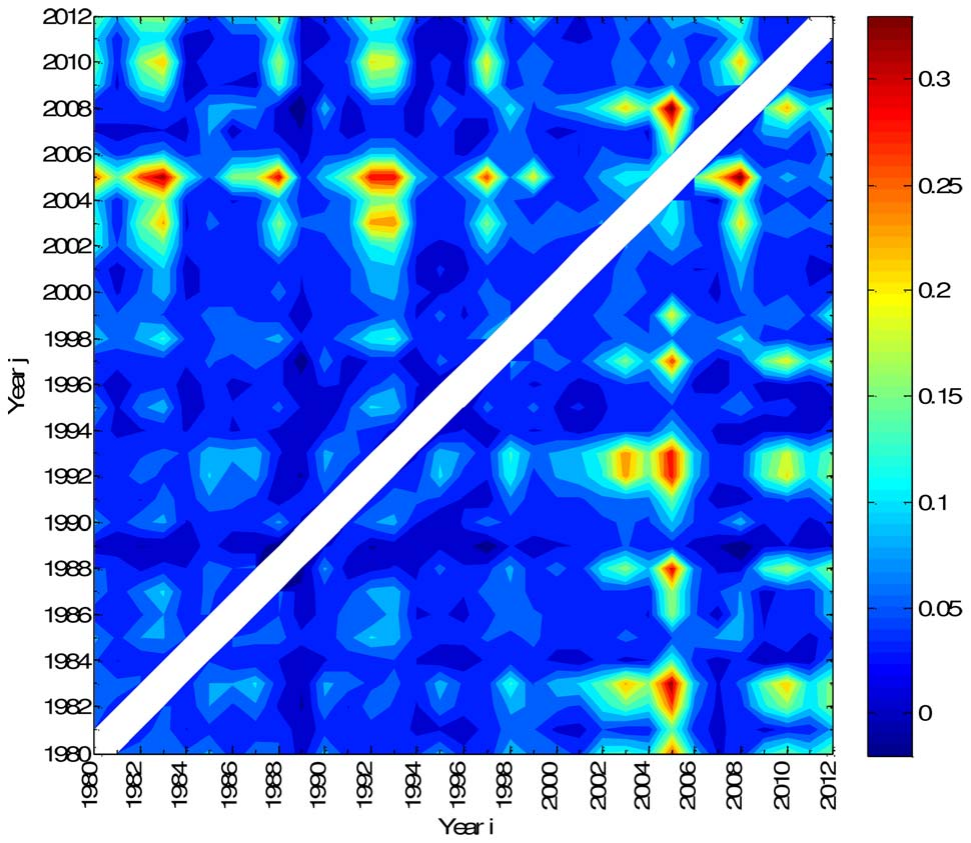
Contour map of the mutual information, *I_N_*(*X_i_*, *X_j_*), between occurrences registered in Portugal during 1980–2012. The cutoff threshold value *A_min_* = 10 ha is adopted.

Fig. 4 depicts in a contour map the mutual information, *I_N_*(*X_i_*, *X_j_*), between every pair of years *i*, *j* = 0, …, 32. The probabilities for calculating the mutual information are estimated from the histograms of amplitudes *A_k_*, constructed considering 476 bins, each one having width equal to 0.1 ha. To facilitate the comparison the cases *i* = *j* (i.e., those with maximum value of mutual information) are removed from the graph.

The map reveals strong correlations between certain years, corresponding to higher values of mutual information. This is well noted for the years a =  {2003, 1983}, b =  {2003, 1993}, c =  {2005, 1980}, d =  {2005, 1983}, e =  {2005, 1988}, f =  {2005, 1993}, g =  {2008, 2005} and h =  {2010, 1983}. Nevertheless, the analysis is not totally assertive and requires multiple comparisons.

### Hierarchical clustering

Having in mind an efficient method to visualize and to compare results, a hierarchical clustering algorithm is adopted, based on the mutual information, *I_N_*(*X_i_*, *X_j_*), between pairs of objects.

**Figure 5:**
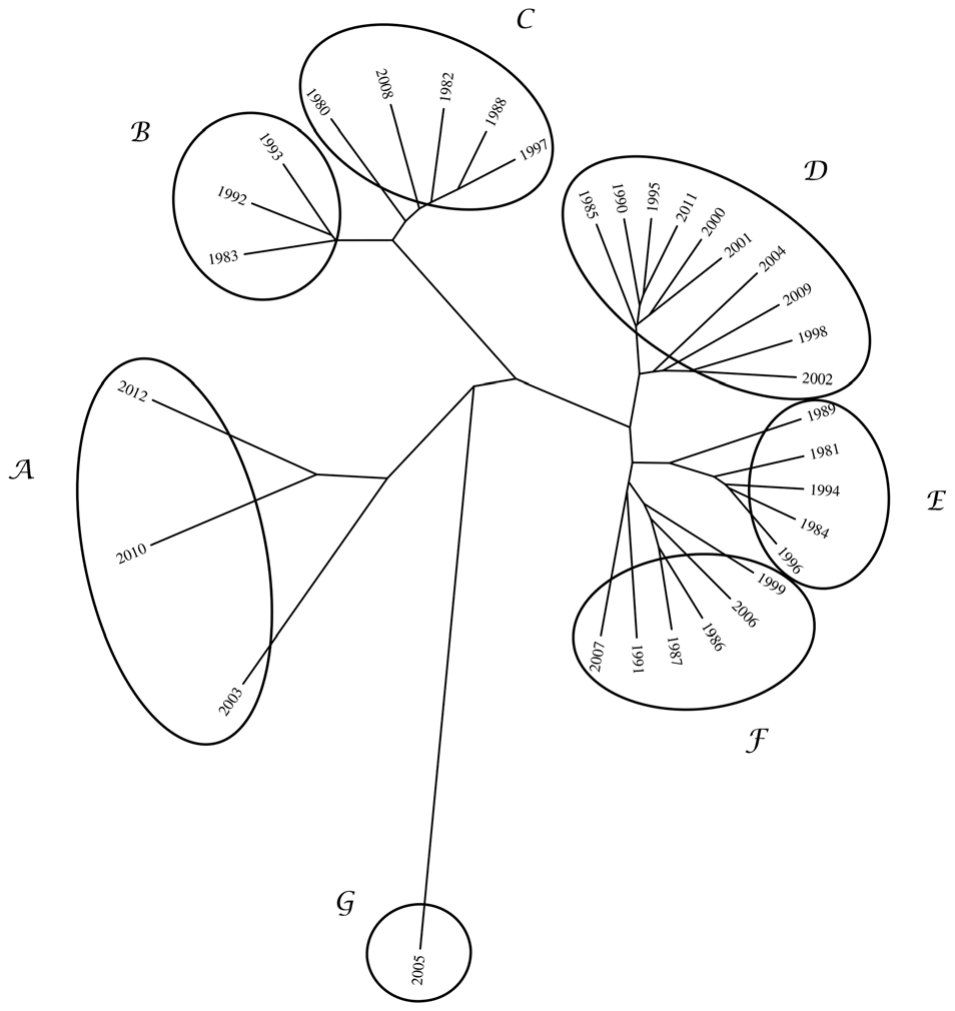
Tree representing mutual information, *I_N_*(*X_i_*, *X_j_*), between occurrences registered in Portugal during 1980–2012. The cutoff threshold value *A_min_* = 10 ha is adopted.

The goal of hierarchical clustering is to build a hierarchy of clusters, in such a way that objects in the same cluster are, in some sense, similar to each other [30], [32]–[33]. Based on a measure of dissimilarity between clusters, those are combined (or, alternatively, split) for agglomerative (or, alternatively, divisive) clustering. This is achieved by using an appropriate metric, quantifying the distance between pairs of objects, and a linkage criterion, defining the dissimilarity between clusters as a function of the pairwise distances between objects. The results of hierarchical clustering are presented in a phylogenetic tree adopting the successive (agglomerative) clustering and average-linkage method (Fig. 5). The software PHYLIP was used for generating both graphs (http://evolution.genetics.washington.edu/phylip.html).

Fig. 5 unveils groups of objects (years) in such a way that objects in the same group (cluster) are more similar to each other than to those in other groups. For example, we can easily identify clusters composed by years A =  {2003, 2010, 2012}, B =  {1983, 1992, 1993} and C =  {1980, 1982, 1988, 1997, 2008,}. On the contrary, year G =  {2005} is far aside, meaning that it is different from all the rest. Both representations of Fig. 4 and Fig. 5 can be used to visualise and to compare the events, in an annual basis. Fig. 5 leads to a result easier to interpret than Fig. 4, as it identifies groups of objects that are similar.

## MDS Analysis and Visualization

In this section we adopt the MDS tools to handle information and the relationships embedded into the data.

MDS is a statistical technique for visualizing data that can reveal similarities between objects. The algorithm requires the definition of a similarity measure (or, inversely, of a distance) and the construction of a *s*×*s* symmetric matrix **D** of similarities (or distances) between each pair of *s* objects. MDS assigns a point to each object in a *m*-dimensional space and arranges the set in order to reproduce the observed similarities. A shorter (larger) distance between two points means that the corresponding objects are more similar (distinct). For *m* = 2 or *m* = 3 dimensions the resulting locations may be displayed in a “map” that can be visualized [34]–[39].

**Figure 6:**
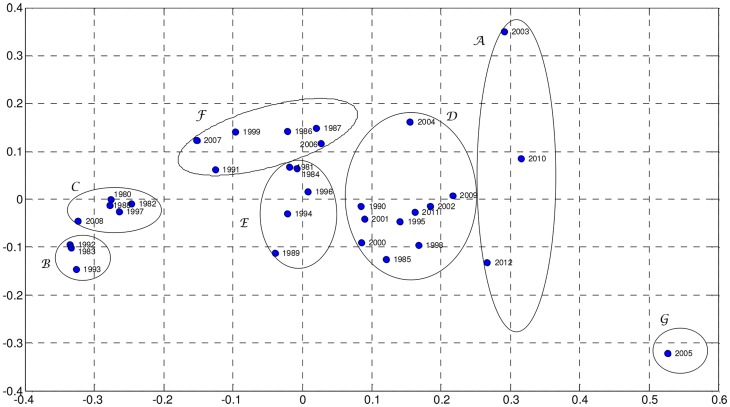
MDS map based on matrix D, for visualization space with dimension *m* = 2.

**Figure 7:**
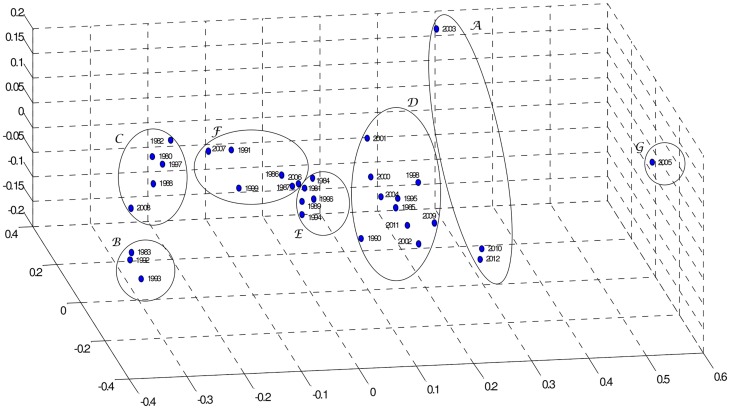
MDS map based on matrix D, for visualization space with dimension *m* = 3.

**Figure 8:**
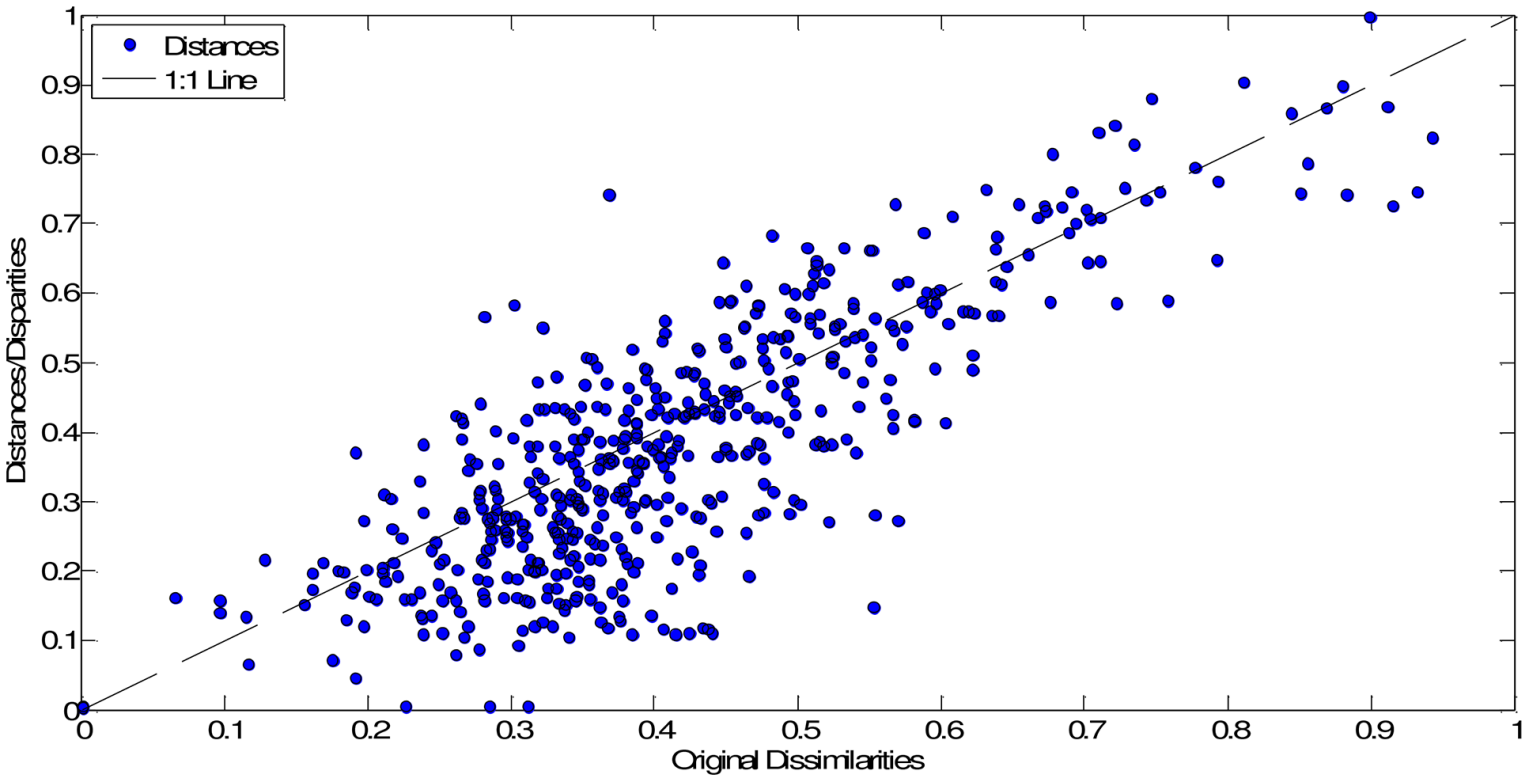
Shepard plot of the MDS map based on matrix D, for visualization space with dimension *m* = 2.

**Figure 9:**
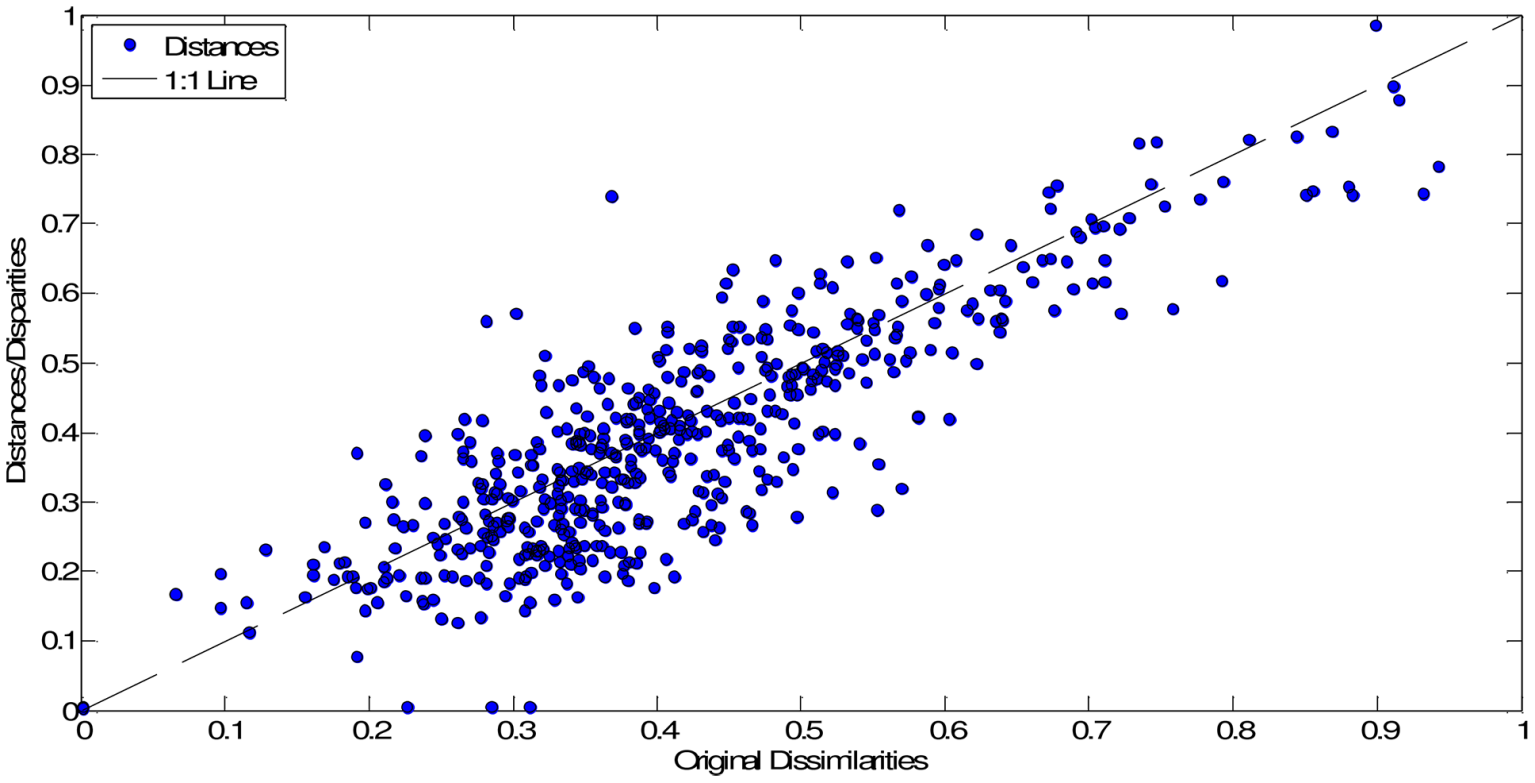
Shepard plot of the MDS map based on matrix D, for visualization space with dimension *m* = 3.

**Figure 10:**
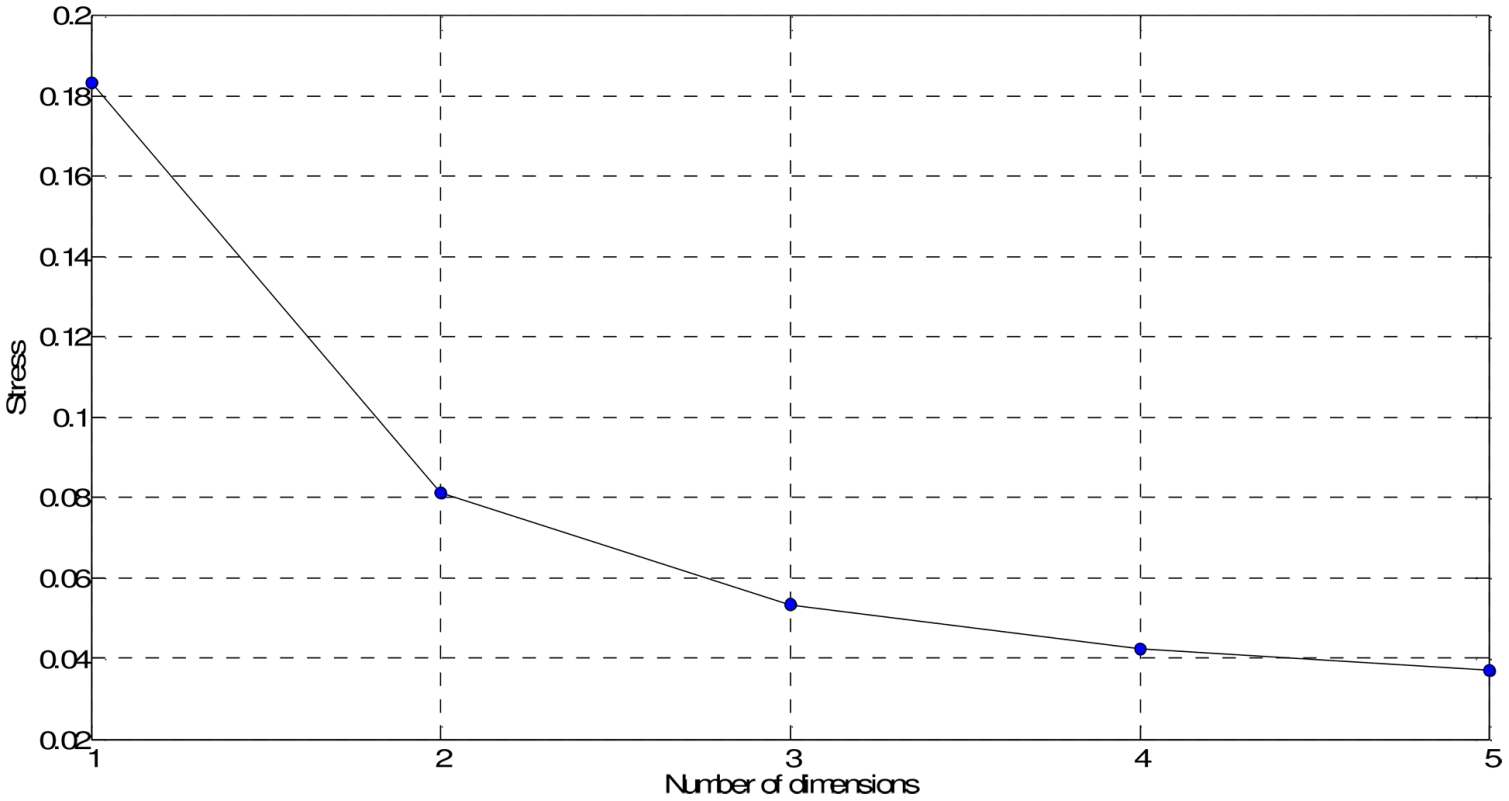
Stress plot for the MDS based on matrix D.

In our case, we obtain **D** (33×33 dimensional) by means of the mutual information (4). Fig. 6 and Fig. 7 show the MDS maps for *m* = 2 and *m* = 3, respectively. The Shepard and the stress plots assess the quality of the MDS maps. The Shepard diagrams (Fig. 8 and Fig. 9) show an acceptable distribution of points around the 45 degree line, which means a good fit of the distances to the dissimilarities. On the other hand, the stress plot reveals that a three dimensional space describes adequately the data (Fig. 10). This can be concluded by observing the stress line, which diminishes strongly until the dimensionality is two, moderately towards dimensionality three and weakly from then on. Often, the maximum curvature point of the stress line is adopted as the criterion for deciding the dimensionality of the MDS map.

The MDS maps of Fig. 6 and Fig. 7 confirm the groups previously identified by the hierarchical clustering and, consequently, the relationships between the corresponding yearly patterns. Comparing Fig. 5 with Fig. 6 and Fig. 7, we conclude that all allow an easy interpretation of the results. The MDS maps, in particular the 3D plot, are more intuitive than the phylogenetic tree. Moreover, most software for MDS analysis allows the user to rotate and visualise the maps from different perspectives, easing the identification of clusters. This is useful especially when dealing with large amounts of data.

## Forest Fires Spatial Patterns

In this section we study forest fires in a complementary line of thought, namely by considering spatial information. First, we divide the geographic territory under study (i.e., 36.95°≤ lat ≤42.15°; −9.50°≤ lon ≤−6.19°), using a *M*×*N* (*M* = 30, *N* = 15) rectangular grid, and we determine the 33 bidimensional histograms of relative frequencies for all years in the period 1980–2012. Second, for characterizing the histograms, we calculate the Shannon entropy, *S_i_*, given by:
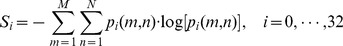
(8)where the probabilities *p_i_*(*m*, *n*) are approximated by the relative frequencies.

**Figure 11:**
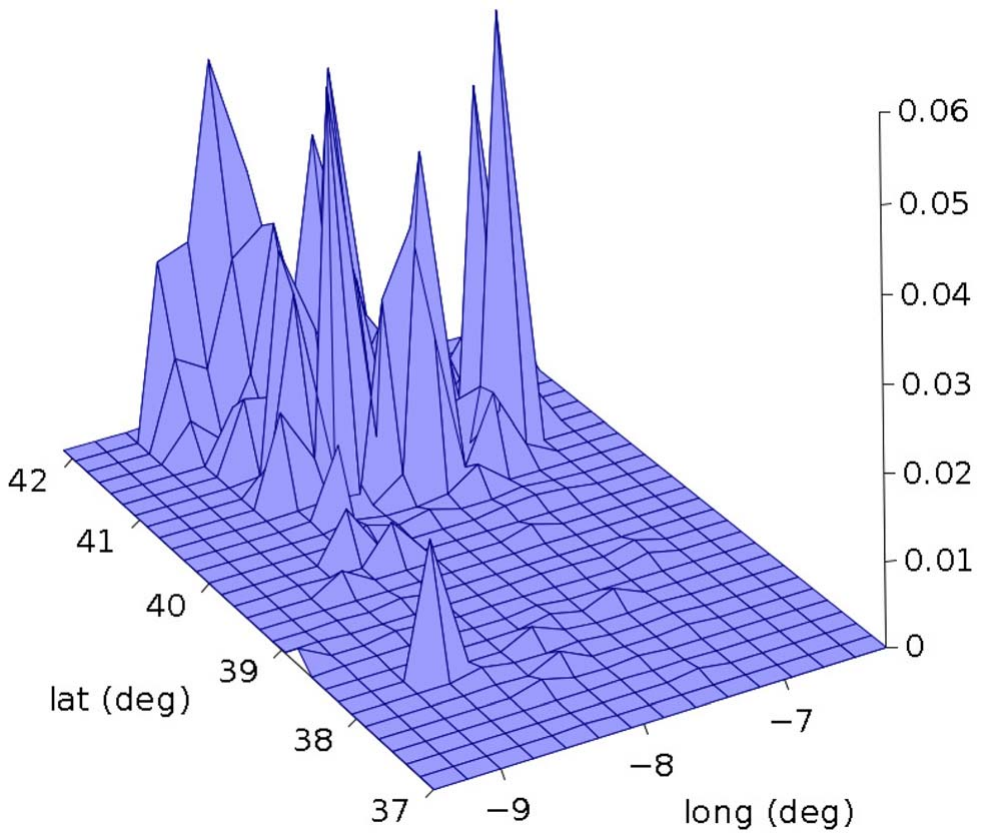
Bidimensional histogram of forest fires versus latitude and longitude, in Portugal, for year 2010.

In Fig. 11, for example, we depict the bidimensional histogram for year 2010. The corresponding entropy is *S_i_* = 4.08 (*i* = 30).

For histogram comparison we calculate a *M*×*N* symmetric matrix **D** =  [*d_ij_*], where
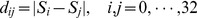
(9)


**Figure 12:**
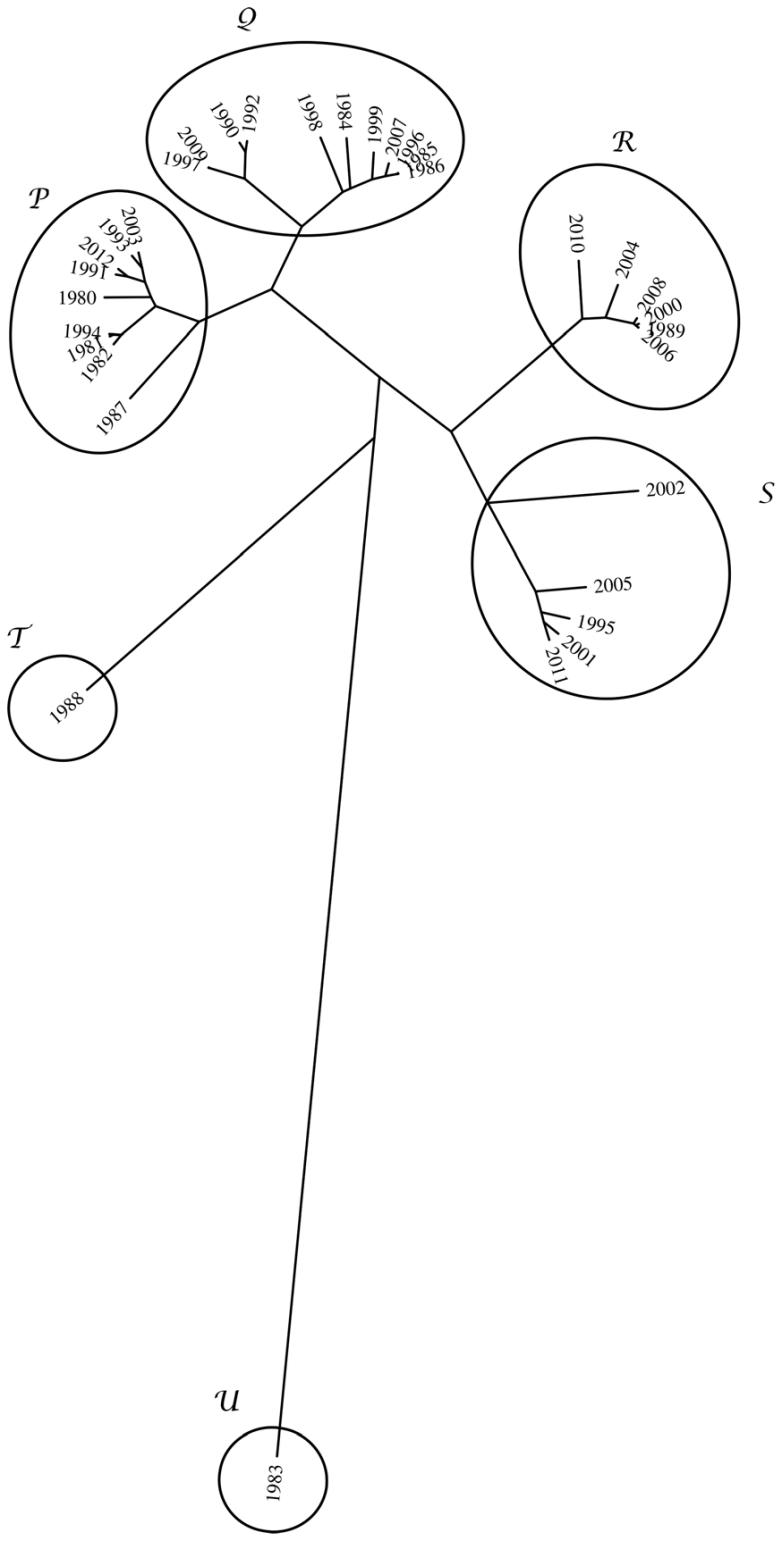
Tree comparing the 33 bidimensional histograms based on index *d_ij_*.

The results are visualized in the phylogenetic tree of Fig. 12. We can observe six clusters: P =  {1980, 1981, 1982, 1987, 1991, 1993, 1994, 2003, 2012}; Q =  {1984, 1985, 1986, 1990, 1992, 1996, 1997, 1998, 1999, 2007, 2009}; R =  {1989, 2000, 2004, 2006, 2008, 2010}; S =  {1995, 2001, 2002, 2005, 2011}; T =  {1983} and U =  {1988}.

**Figure 13:**
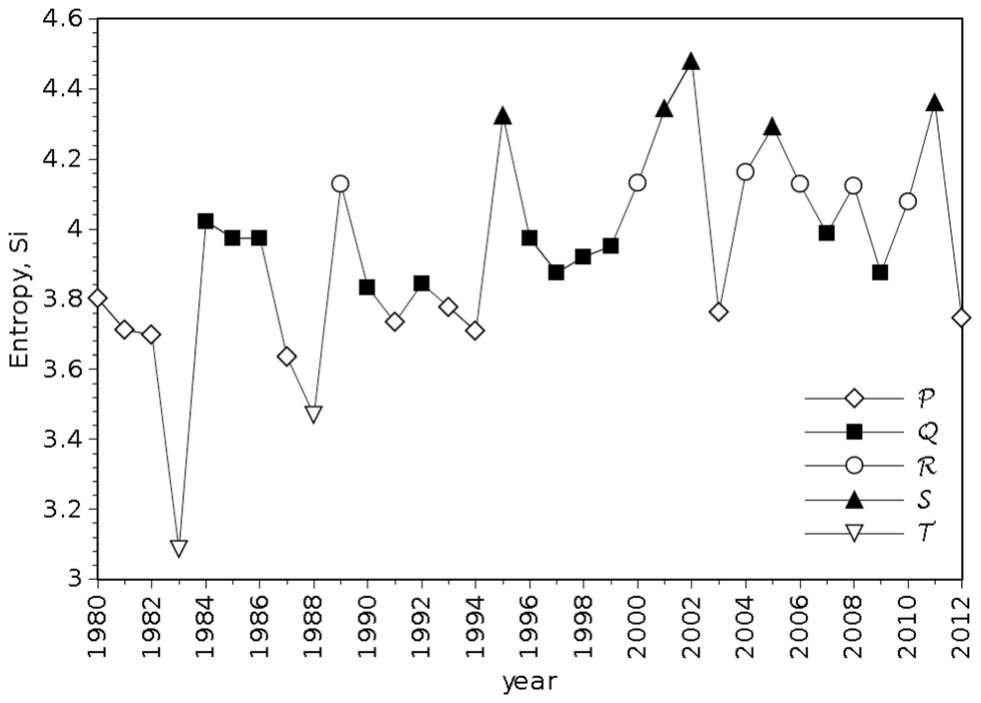
Entropy, *S_i_*, versus year, during the time period 1980–2012.

The evolution of *S_i_* versus year is represented in Fig. 13, where the clusters shown in Fig. 12 are identified. In this chart is clear a large volatility and apparently some increase of entropy along time.

In a more global perspective, we verify that amplitude and space data lead to distinct observations. The conclusions are ‘decoupled’ and reveal that both directions must be explored, with more data, in order to include all information in a global tool of analysis.

**Figure 14:**
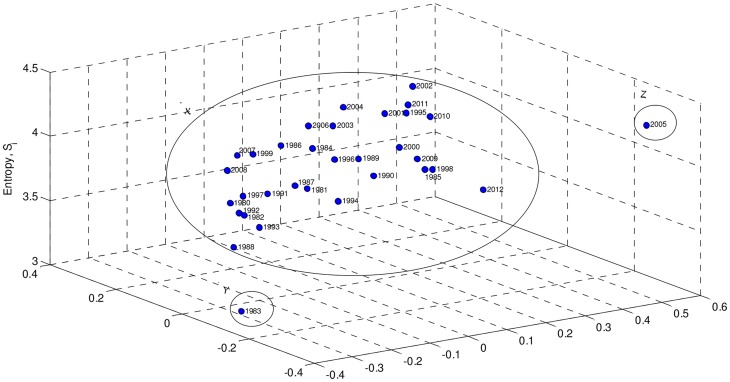
MDS 2D plot with the vertical axis representing Shannon entropy.

In this line of though, we embed amplitude and space data into a single graph by adding to the bidimensional MDS plot of Fig. 6 a vertical axis representing the Shannon entropy (Fig. 14).

We note that only two years, Y =  {1983} and Z =  {2005}, have now a clearly distinct separation from the main cluster, X. In Fig. 13 we observed them to be located at near extreme values, but, as mentioned, it is difficult to get idea due to large volatility. The embedding of amplitude-space techniques produced a clear classification pattern.

## Conclusions

We analysed forest fires from the perspective of dynamical systems. Data from a public domain forest fires catalogue, containing information of events for Portugal, during the period 1980–2012, was studied in an annual basis. Mutual information to correlate annual patterns was considered. Phylogenetic trees generated by hierarchical clustering algorithms and MDS visualization tools were used to compare to extract relationships among the data and to identify forest fire patterns. Those tools allow different perspectives over forest fires that may be used to better understand the dynamics emerging in the plethora of phenomena that occur in forest fires.
